# The association between lipid profile, oxidized LDL and the components of metabolic syndrome with serum mineral status and kidney function in individuals with obesity

**DOI:** 10.1186/s13104-023-06472-2

**Published:** 2023-09-05

**Authors:** Abnoos Mokhtari Ardekani, Zahra Hamidi Nava, Burhan Abdullah Zaman, Sahar Vahdat, Amir-Hossein Lame-Jouybari, Azam Mivefroshan

**Affiliations:** 1 Endocrinology and Metabolism Research Center, Institute of Basic and Clinical Physiology Science, & Physiology Research Center, Kerman University of Medical Sciences, Kerman, Iran; 2 Department of Internal Medicine, Faculty of Medical Science, Mazandaran University of Medical Science, Sari, Iran; 3 Medical Physiology, Basic Sciences Department, College of Pharmacy, University of Duhok, Kurdistan, Iraq; 4 Isfahan Kidney Diseases Research Center, School of Medicine, Khorshid Hospital, Isfahan University of Medical Sciences, Isfahan, Iran; 5 Student Research Committee, Tabriz University of Medical Sciences, Tabriz, Iran; 6 Department of Internal Medicine, School of Medicine, Urmia University of Medical Sciences, Urmia, Iran

**Keywords:** Metabolic syndrome, Obesity, Mineral hemostasis, Renal function

## Abstract

**Background:**

Metabolic syndrome (MetS) is presented with a cluster of cardio-metabolic risk factors with widespread prevalence. In the present case-control study, we aimed to examine the relationship between several minerals and renal function tests with the components of MetS in individuals with obesity.

**Methods:**

This study included 127 individuals with obesity of both gender with or without MetS as the case and control, respectively. MetS was characterized based on the Adult Treatment Panel III (ATP III) criteria. Anthropometric variables and blood pressure were recorded. Mineral status including serum magnesium, copper, calcium, phosphorous, and iron were measured using standard colorimetric methods. Also, the serum lipid levels, concentrations of oxidized low-density lipoprotein (Ox-LDL), and renal function tests, including total protein, albumin, urea, creatinine, and uric acid were evaluated using commercial enzyme-linked immunosorbent assay (ELISA) kits.

**Results:**

According to our results, individuals with obesity and MetS had higher levels of waist circumference (WC) and diastolic blood pressure (P < 0.05) compared to individuals with obesity and without MetS. Moreover, individuals with obesity and MetS had higher levels of serum total cholesterol (TC), triglyceride (TG), insulin, and iron (P < 0.05). In individuals with obesity and MetS, iron and albumin showed a positive relationship with LDL cholesterol and TG concentrations, respectively (P < 0.05 for all of them). Also, there was a positive association between serum magnesium and Ox- LDL in individuals with obesity with MetS. While, in individuals with obesity and without MetS, only a positive association between urea and uric acid with WC was observed (P < 0.05).

**Conclusion:**

Our results suggest that disturbed serum lipids in obesity-metabolic syndrome is associated with homeostatic changes in the level of minerals or proteins that are involved in their metabolism. Although, further studies are needed to better explain and clarify the underlying mechanism of observed relationships.

**Supplementary Information:**

The online version contains supplementary material available at 10.1186/s13104-023-06472-2.

## Background

Metabolic syndrome (MetS) is characterized by various risk factors such as hypertension, central obesity, glucose intolerance, insulin resistance (IR), and dyslipidemia [1–3]. It is highly prevalent worldwide, with a prevalence ranging from 20 to 25% in the adult population [4–7]. In Iran, a high prevalence of MetS has been recognized. According to the results of a recent meta-analysis study, the prevalence among the Iranian population is 26% [8]. Several studies have revealed that not only MetS increases the risk of cardiovascular disease, but also the incidence of type 2 diabetes mellitus (T2DM) and chronic kidney disease (CKD) in the general population [9–12].

The relationship between MetS and CKD is garnering increased attention in medicinal research. Several studies have demonstrated the association between MetS and CKD indicators such as microalbuminuria, proteinuria, glomerular filtration rate, interstitial fibrosis, and tubular atrophy [13–15]. Hyperinsulinemia in MetS, results in excessive renal sodium maintenance and increased blood pressure [16, 17], which in turn can cause several renal diseases by activation of the renin-angiotensin-aldosterone system, adipokine imbalance, and endothelial dysfunction [13, 14].

Dietary minerals such as magnesium, copper, calcium, phosphorous, and iron have a potential role in growth, immunity, neurological functions and metabolism in the human body and are hypothesized to be involved in diabetes and MetS [18–22]. Some of these minerals have an important role in the pathogenesis of metabolic syndrome; several studies have revealed that metabolic syndrome is associated with lower serum magnesium concentrations and that magnesium supplementation will be effective to compensate for this deficiency [23, 24]. Also, copper deficiency in metabolic syndrome and its role in the development of coronary heart disease (CHD) in these patients [25, 26]. Evidence has emphasized the particular role of excess iron and iron overload in the incidence of metabolic disorders like non-alcoholic fatty liver disease or heart attacks in patients with MetS [27, 28]. And this is also true about calcium-phosphorous products and the incidence of coronary artery disease (CAD) in patients with MetS [29].

Moreover, clinical symptoms of MetS might also be indirectly or directly associated with changes in the metabolism of minerals [19, 20]. IR or oxidative stress might also be associated with mineral metabolism [30]. Taking MetS and minerals into consideration, dietary consumption of copper and iron was related to an increased risk of MetS [19, 28, 31]. Song et al. revealed an inverse association between dietary magnesium intake and MetS prevalence among healthy American women [32]. Moreover, as reported previously, excess body iron is possibly associated with the development of MetS and T2DM [33, 34]. There is little evidence about the relationship between serum levels of minerals and MetS, and most of the existing findings are not statistically significant [35, 36].

MetS is associated with many comorbidities such as chronic kidney disease (CKD). This association might be through mechanisms related to excessive renal perfusion and hyperfiltration [37, 38]. Lea et al. revealed that every single component of MetS may not have a serious effect on kidney disease, but the combination of components might alter kidney function [39]. Inconsistent with this finding, in a Southeast Asian Cohort study, among five MetS components, high blood pressure was related to the higher prevalence of CKD [40]. In one study by Maric C et al., high blood pressure and slight hyperglycemia were associated with an increased risk of CKD and microalbuminuria [41]. Prior studies have reported a relationship between MetS and renal disease, defined as low creatinine clearance or proteinuria and increased risk for micro-albuminuria, a marker of kidney disease [37, 42]. This highlights the importance of the link between MetS and mineral status and kidney function.

Several pieces of evidence have emphasized the importance of maintaining adequate levels of these minerals in the prevention and management of MetS and its associated complications; in the current case-control study, we aimed to investigate the association between mineral status and renal function tests with MetS components in individuals with obesity and MetS compared with individuals with obesity but without MetS.

## Materials and methods

### Study design

This case-control study included 62 individuals with obesity and MetS and 65 individuals with obesity and without MetS from August to October 2020. The sample size was obtained based on the results of a previous study [43], considering a power of 80%, and a type I error of 5% using power analysis and sample size software (Statistica software, version 10). There were 62 cases (individuals with obesity and MetS) and 65 control samples (individuals with obesity and without MetS) with an approximately 1:1 ratio. Groups were matched based on gender, body mass index (BMI), and age. The subjects were recruited with public announcement and the randomization procedure was used to avoid any selection or volunteer bias. In this procedure, the volunteers that met our inclusion criteria were assigned each a random number. Then, study participants were chosen randomly based on these numbers.

Inclusion criteria were as follows: individuals with obesity and with or without MetS, aged 20–50 years, and BMI ≥ 30 kg/m^2^. The exclusion criteria were: participants that used vitamin and mineral supplements in the last three months; those with any type of diabetes, bone problems or osteopenia/osteoporosis, those who were under hormonal drugs, antidepressants, glucocorticoids, anti-diuretics, antibiotics medications; patients with any history of atherosclerosis, cancer, chronic liver disease, kidney diseases, acute infections, and recent acute illness; menopausal, pregnant, and lactating women. Patients were informed about the study aims and completed a written informed consent before participating in the study.

### Anthropometric measurements

Body weight was measured at fasting state by calibrated Seca scale (Dubai, United Arab Emirates) with light clothes and without shoes and an accuracy of 100 g, and height was measured using stadiometer at approximately 0.5 cm. A non-stretchable tape was used to measure waist circumference (WC) at the narrowest area of the waist approximately 0.5 cm. BMI was defined as weight divided by height squared and obesity was defined as BMI more than 30 kg/m ^2^.

### Appetite measurements

The Visual Analogue Scale (VAS) was used for appetite assessments. This questionnaire includes questions about satiety, desire to eat sweet/salty/fatty foods, hunger, fullness, and prospective food intake. Individuals were asked to mark their senses on a 100 mm line and quantification of appetite was done by measuring the distance from the beginning to the marked point (Supplementary material 1) [44].

### Criteria of metabolic syndrome (MetS)

The MetS was defined according to the criteria of the Adult Treatment Panel III (ATP III) [45] as the presence of three or more of the following characteristics: (a) WC ≥ 102 cm for men and ≥ 88 cm for women, (b) serum HDL-C ≤ 40 mg/dl for men and ≤ 50 mg/dl for women, (c) serum TG ≥ 150 mg/dl, (d) fasting blood glucose (FBG) ≥ 100 mg/dl, and (e) hypertension (systolic blood pressure (SBP) ≥ 130 mmHg or diastolic blood pressure (DBP) ≥ 85 mmHg that includes the Stage 1 of HTN as blood pressure ≥ 130 and ≤ 139/≥80 and ≤ 89 mm Hg and Stage 2 HTN as blood pressure ≥ 140/≥90 mm Hg [46].

### Biochemical assessments and blood pressure

In this study, 5 ml of fasting blood samples were collected from all participants after 12–14 h of fasting. Serum was extracted from blood samples by centrifugation at 2500 rpm at room temperature for 10 min (Beckman Avanti J-25, USA) and immediately transferred to -70^°^C until assay. Mineral status, including serum magnesium, copper, calcium, phosphorous, and iron was measured using standard colorimetric methods (kits from Ziest Chem Co., Tehran-Iran). The intra- and inter-assay coefficients of variation (CVs) were 0.92 and 1.09 for magnesium; 1.9 and 6.9 for copper; 1.4 and 2.7 for calcium; 1.9 and 3.1 for phosphate; and < 10 for iron, respectively. Kidney function tests including blood creatinine (Man company, Cat No: 613,027, Iran. Intra- and inter-assay CVs were < 3.5% both), urea (Man company, Cat No: 613,020, Iran. Intra- and inter-assay CVs were 1.1 and 1.4 respectively), uric acid (Man company, Cat No: 613,028, Iran. Intra- and inter-assay CVs were 1.3 and 1.6 respectively) albumin (Man company, Cat No: 613,040, Iran Intra- and inter-assay CVs were 2 and 1.8 respectively), and total protein (Bioassay Technology Laboratory, Shanghai Korean Biotech, Shanghai City, Cat No: E1149Hu, China. Intra- and inter-assay CV were 1.5 and 2.6 respectively), serum FSG (glucose oxidase, phenol, 4-amino antipyrine peroxidase method, Pars Azmoon, Cat No: 1,500,017, Karaj, Iran. Intra- and inter-assay CV were 1.63 and 2.2 respectively), were measured using the commercial kits .

Serum Ox-LDL was measured by Bioassay Technology Laboratory ELISA kit with the intra-assay (CV of < 8%) and inter-assay (CV of < 10%). Serum insulin level was evaluated by ELISA kit (DiaMetra, Milano, Italy) with the intra-assay (CV of ≤ 5%) and inter-assay (≤ 10%). Furthermore, serum lipid profiles, including TG (kit No. 1,500,032), TC (kit No. 1,500,010), and HDL-C (kit No. kit No. 0121500) were assessed by Abbott ALCYON™ 300 using ELISA kits (Pars Azmoon, Tehran, Iran, kit No. 031 500). The mean intra- and inter-assay CVs were 1.53 and 1.60 for TG; < 4 for HDL-C; and 0.061 and 1.14 for TC, respectively. All these assessments were done in a blinding status to group assignments. Blood pressure was measured with a standard mercury sphygmomanometer twice in the same arm after at least 15 min of rest and the mean of the two measurements was used for analysis. All the tests were conducted according to the directions of the manufacturers.

### Statistical analyses

SPSS software (version 24, SPSS Inc., USA) was used for statistical analyses. We reported quantitative data as mean ± standard deviation and qualitative variables were reported as frequency (%). Independent sample t-test and partial correlation analysis were used for data analysis, and the effects of confounders, including age, gender, and BMI were adjusted. P-values less than 0.05 were considered significant.

## Results

The present study included 127 individuals with obesity (62 with MetS and 65 without MetS).

**The results are as follows**:

### Demographic findings of the study subgroups

Demographic information of the participants is provided in Table 1. WC (P = 0.02) and DBP (P = 0.02) were significantly lower in subjects without MetS compared to those with MetS. No significant difference was observed between the groups regarding gender, age, BMI, and SBP (P > 0.05). Appetite was non-significantly higher among obese adults with MetS compared with obese adults without MetS (P = 0.35).

**Table 1:** The baseline characteristics of the study subjects

Variables	The study subgroups		P-value
	Obese adults without MetS (n = 62)	Obese adults with MetS (n = 65)	
Age (Year)	35.46 ± 8.01	32.15 ± 9.97	0.33*
Gender			
Female [n(%)]	15 (24)	17 (26)	0.21**
Male [n(%)]	47 (76)	48 (74)	
Appetite	190.00 ± 34.01	204.23 ± 46.43	0.36*
WC	95.13 ± 7.01	102.07 ± 8.55	0.02*
BMI (kg/m2)	32.60 ± 2.49	33.70 ± 3.51	0.34*
SBP (mmHg)	122.00 ± 6.76	126.10 ± 9.60	0.19*
DBP (mmHg)	79.33 ± 4.57	83.84 ± 5.06	0.02*

*P-value was reported based on Independent Sample T test. ** P-value was reported based on Chi-square test. BMI: Body Mass Index; SBP: Systolic Blood Pressure; DBP: Diastolic Blood Pressure; MetS: Metabolic Syndrome. Data are presented as mean ± SD. WC and DBP are significantly higher among obese adults with MetS. No significant difference was observed for other variables.

### Biochemical variables in study subgroups

Table 2 presented the biochemical values, including mineral status and renal function test compared between the two groups. Individuals with obesity and MetS represented significantly higher levels of serum TC, TG, insulin, and iron, but lower uric acid values (P < 0.05 for all of them).

**Table 2:** The comparison of biochemical parameters among study groups

Variables	The study sub-groups		P-value*
	Obese adults without MetS (n = 62)	Obese adults with MetS (n = 65)	
TG (mg/dl)	128.46 ± 57.67	186.08 ± 73.65	0.028*
HDL (mg/dl)	34.00 ± 11.26	31.61 ± 8.62	0.540*
LDL (mg/dl)	115.82 ± 24.65	126.33 ± 27.28	0.134*
Total cholesterol (mg/dl)	177.13 ± 31.81	195.69 ± 31.40	0.022*
FSG (mg/dl)	89.22 ± 9.16	99.88 ± 35.95	0.33
Insulin (μIU/l)	5.77 ± 2.42	11.96 ± 9.40	0.017*
Total Protein (g/dl)	9.68 ± 2.51	8.27 ± 3.11	0.12
Albumin (g/dl)	5.94 ± 1.60	5.80 ± 1.61	0.78
Urea (mg/dl)	17.75 ± 5.56	15.95 ± 4.53	0.30
Creatinine (mg/dl)	2.05 ± 0.60	2.17 ± 0.83	0.59
Uric acid (mg/dl)	5.77 ± 1.78	4.67 ± 1.69	0.049*
Magnesium (mg/dl)	1.81 ± 0.18	1.87 ± 0.33	0.38
Copper (μg/dl)	91.54 ± 21.51	90.45 ± 19.23	0.32
Iron (mcg/dL)	68.3 ± 18.28	75.35 ± 22.83	0.047*
TIBC (μg/dl)	291.26 ± 19.81	299.75 ± 13.17	0.18
Calcium (mg/dl)	8.61 ± 0.63	8.81 ± 0.64	0.22
Phosphorus (mg/dl)	4.45 ± 2.20	3.71 ± 1.11	0.29

*P-value was reported based on Independent Sample T test and the values less than 0.05 were considered as statistically significant. TG: triglyceride; HDL: high density lipoprotein; LDL, low density lipoprotein; FSG, fasting serum glucose; TIBC, total iron binding capacity. TG, total cholesterol, insulin and iron concentrations were significantly higher among obese adults with MetS. While obese adults without MetS had higher uric acid values.

### The association between MetS components, mineral status and kidney function tests

The association between mineral statuses with the components of MetS is shown in Table 3. In individuals with obesity and MetS, serum magnesium was in positive association with ox-LDL (r = 0.44; P = 0.037) and serum iron was positively associated with LDL (r = 0.48; P = 0.05). No significant association between serum minerals and MetS components was observed in individuals with obesity but without MetS and also in combined groups. Table 4 represents the association between kidney function tests and components of MetS. In individuals with obesity and MetS, albumin showed a positive relationship with TG concentrations (r = 0.49; P = 0.04). While in individuals with obesity but without MetS, urea (r = 0.86; P = 0.05) and uric acid (r = 0.88; P = 0.04) were positively associated with WC. Furthermore, in combination of both groups, albumin was positively associated with TG (r = 0.45, P = 0.02), and negatively associated with HDL-C (r= -0.41; P = 0.04), while uric acid was positively associated with WC values (r = 0.43; P = 0.03).

**Table 3:** The correlation between magnesium, copper, calcium, phosphorous, and iron with components of metabolic syndrome in study groups

Study groups		Variables									
		Magnesium		Copper		Calcium		Phosphorous		Iron	
	Variables	r	P	r	P	r	P	r	P	r	P
Obese adults with MetS	TC	0.03	0.9	-0.04	0.86	-0.23	0.37	0.1	0.68	-0.27	0.31
	TG	-0.37	0.15	0.34	0.19	-0.37	0.15	0.18	0.5	0.24	0.35
	HDL	0.35	0.17	-0.15	0.57	0.18	0.48	0.09	0.72	-0.02	0.92
	WC	0.33	0.2	-0.34	0.19	-0.04	0.87	-0.25	0.34	-0.32	0.22
	LDL	-0.05	0.84	-0.35	0.17	-0.3	0.24	0.2	0.45	**0.48**	**0.049**
	OX-LDL	**0.44**	**0.037**	-0.05	0.84	0.16	0.53	-0.45	0.07	-0.26	0.33
Obese adults without MetS	TC	0.38	0.61	-0.01	0.98	0.1	0.9	-0.04	0.96	-0.09	0.9
	TG	0.25	0.75	0.64	0.35	-0.48	0.52	-0.71	0.28	-0.9	0.1
	HDL	-0.88	0.12	0.2	0.79	-0.38	0.61	-0.1	0.9	0.46	0.53
	WC	0.22	0.77	0.19	0.8	-0.1	0.89	-0.23	0.76	-0.64	0.34
	LDL	0.46	0.53	-0.36	0.63	0.41	0.58	0.32	0.67	0.22	0.77
	OX-LDL	-0.79	0.2	0.01	0.98	-0.2	0.79	0.09	0.91	0.76	0.23
Combination of the two groups	TC	-0.05	0.79	0.1	0.64	-0.16	0.47	0.008	0.97	-0.23	0.28
	TG	-0.1	0.65	0.06	0.77	-0.19	0.38	-0.06	0.76	0.08	0.71
	HDL	0.15	0.5	0.02	0.92	0	0.99	0.09	0.68	-0.04	0.84
	WC	0.001	0.99	-0.005	0.98	-0.12	0.56	-0.29	0.19	-0.36	0.09
	LDL	-0.02	0.9	-0.1	0.63	-0.1	0.63	0.17	0.44	-0.34	0.11
	OX-LDL	0.28	0.2	-0.04	0.83	0.04	0.84	-0.21	0.33	-0.16	0.45

Data analysis was done by partial correlation. MetS: Metabolic Syndrome; SBP: Systolic Blood Pressure; DBP: Diastolic Blood Pressure; TG: triglyceride; HDL: High-Density Lipoprotein; WC: Waist Circumference; LDL: Low-Density Lipoprotein. In obese adults with MetS, ox-LDL was positively associated with serum magnesium and iron concentrations. No other association was observed for obese adults without MetS or the combination of both groups.

**Table 4:** The correlation between total protein, albumin, urea, creatinine, and uric acid with the components of metabolic syndrome in study groups

Study groups		Variables									
		Total Protein		Albumin		Urea		Creatinine		Uric acid	
	Variables	r	P	r	P	r	P	r	P	r	P
Obese adults with MetS	TC	-0.21	0.93	-0.19	0.45	-0.13	0.6	-0.14	0.58	0.17	0.49
	TG	0.29	0.24	**0.49**	**0.04**	-0.18	0.48	0.17	0.5	0.27	0.91
	HDL	-0.1	0.68	-0.41	0.1	0.38	0.13	0.23	0.36	0.27	0.28
	WC	-0.25	0.31	-0.36	0.15	-0.07	0.97	-0.15	0.55	0.28	0.27
	LDL	-0.12	0.64	0.34	0.17	-0.03	0.88	-0.05	0.84	0.33	0.18
	OX-LDL	-0.12	0.64	-0.19	0.44	-0.39	0.11	-0.13	0.61	-0.2	0.44
Obese adults without MetS	TC	0.41	0.48	-0.61	0.27	0.03	0.95	0.25	0.68	-0.45	0.44
	TG	-0.14	0.82	0.39	0.51	0.18	0.77	0.06	0.91	-0.005	0.99
	HDL	0.15	0.8	-0.43	0.46	0.18	0.76	-0.39	0.5	0.48	0.4
	WC	0.63	0.24	0.3	0.61	**0.86**	**0.049**	0.41	0.48	**0.88**	**0.04**
	LDL	0.46	0.42	-0.7	0.18	-0.1	0.87	0.34	0.57	-0.58	0.3
	OX-LDL	-0.77	0.12	-0.36	0.54	-0.8	0.1	-0.84	0.07	-0.41	0.48
Combination of the two groups	TC	0.03	0.89	-0.26	0.21	-0.17	0.41	-0.012	0.57	0.06	0.77
	TG	0.003	0.99	**0.45**	**0.02**	0.001	0.99	0.17	0.4	-0.09	0.64
	HDL	0.1	0.63	**-0.41**	**0.04**	0.25	0.22	0.06	0.76	0.38	0.06
	WC	-0.03	0.87	-0.14	0.5	0.08	0.68	-0.09	0.66	**0.43**	**0.03**
	LDL	-0.03	0.86	0.14	0.5	-0.14	0.51	-0.02	0.89	0.15	0.47
	OX-LDL	-0.01	0.95	-0.24	0.24	-0.37	0.07	-0.15	0.46	-0.09	0.66

Data analysis was done by partial correlation. MetS: Metabolic Syndrome; SBP: Systolic Blood Pressure; DBP: Diastolic Blood Pressure; TG: triglyceride; HDL: High-Density Lipoprotein; WC: Waist Circumference; LDL: Low-Density Lipoprotein. In obese individuals with MetS, albumin was in a positive relationship with TG. In obese individuals without MetS, urea and uric acid were positively associated with WC. In combination of both groups, albumin was positively associated with TG, and negatively associated with HDL-C, while uric acid was in positive association with WC values.

## Discussion

In the current study, we evaluated the association between metabolic risk factors, including Ox-LDL, lipid profile and components of MetS with mineral status and renal function tests among individuals with obesity and with or without MetS. We found higher levels of serum TC, TG, insulin, and iron in individuals with obesity and MetS (Table 2). In subjects with MetS, iron and albumin showed a positive relationship with LDL-C and TG concentrations, respectively. Also, a positive association was observed between urea and uric acid values with WC in subjects without MetS. In both groups, albumin was positively associated with TG and negatively associated with HDL-C; moreover, uric acid was positively associated with WC. In addition, serum magnesium was non-significantly higher among the MetS group, and serum magnesium values had a relatively strong significant association (r = 0.44; P = 0.037) with Ox-LDL-C concentrations (Tables 3 and 4).

A cross-sectional study by Xie et al. [47] showed that MetS was independently related to CKD, especially the components of raised TG, FBG, and blood pressure. Similar to our results, in a study that was carried out by Gohari-Kahou et al., there was no significant difference between serum magnesium levels among individuals with MetS and without MetS, and no significant relationship was detected with MetS components [48]. In another cross-sectional study among 11,686 women, an inverse association was reported between MetS prevalence and dietary magnesium intake [32]. Many studies have established a positive association between low dietary magnesium consumption and MetS risk independently from other risk factors, such as BMI, gender, age, exercise, energy, and alcohol intake [49, 50]. Several studies highlighted the role of magnesium as a cofactor of several enzymes involved in the regulation of cellular glucose metabolism [51, 52] and interactions with cellular calcium homeostasis improving insulin sensitivity [53, 54]. It should be noted that serum magnesium does not necessarily reflect body magnesium stores as do intracellular magnesium measurements and is also a poor indicator of intracellular magnesium level. Evidence suggests that magnesium can affect body weight, but the underlying mechanism is still unknown. The hypothesis is that magnesium cooperates with fatty acids to form soaps in the intestine, thus reducing obtained energy from fats [52]. The results of some studies showed that, in magnesium deficiency, serum HDL-C levels were significantly lower and TG levels were significantly higher [55–57]. Magnesium intake may likely contribute to increasing lipoprotein lipase activity [58, 59].

In our study, serum iron concentration was significantly associated with LDL-C levels in individuals with obesity and MetS (Table 3). The suggested mechanism was that iron-induced IR, which in turn led to unfavorable LDL-C, and hepatic IR increased the secretion and synthesis of apoB by protein-tyrosine phosphatase 1B [60]. Consistent with previous studies, body iron stores were associated with MetS components [61–63]. Similar to our results, in a cross-sectional study conducted on 7,540 adults, after adjusting for covariates, the levels of LDL-C were positively related to iron levels [64]. The results of another cross-sectional study showed that serum iron concentration was positively associated with HDL-C, LDL-C, and TC, but was inversely associated with TG in female students [65].

The level of serum iron does not necessarily show a lack of iron stores. While serum iron is commonly used as an indicator of efficient iron, serum ferritin is a more sensitive indicator of iron stores. Caution is necessary when assessing patients with metabolic disorders as a low serum iron may not represent iron insufficiency. In our study, individuals with obesity and MetS showed higher serum iron levels compared to the non-MetS group (Table 2). Iron affects insulin secretion and synthesis in the pancreatic gland and leads to metabolic abnormalities that may augment the production of free radicals [66, 67]. Similar to our results, in a cohort study in Japan, Honda et al. did not find a relationship between iron concentrations and WC in 2,322 CKD patients [68]. However, In a study with 1,130 participants, Choma et al. showed that BMI was inversely related to ferritin level, but WC was positively and non-significantly related to ferritin level [69].

In our findings, serum copper was not significantly different between the two groups, and the association between serum copper and the components of MetS was not significant. Lima et al. [70] indicated a negative correlation between serum copper with LDL-C and TC. However, Obeid et al. reported a positive association between serum copper with TC, LDL-C, and HDL-C levels [25]. These inconsistencies might be due to the difference in general characteristics of study participants, as well as the difference in measurement tools.

In the current study, no significant difference was reported between the two groups in serum calcium. Also, there was no significant association between serum calcium and metabolic risk factors (Tables 3 and 4). Contrary to our findings, a cross-sectional study showed that serum calcium level was related to all the MetS components except HDL-C [71]. However, some previous studies demonstrated that an increased level of intracellular calcium exerts its effects by reducing the number of glucose receptors (e.g. glucose transporter (GLUT) − 4) and reduces insulin receptor’s activity [72, 73]. In a study in Canada, altered calcium homeostasis was associated with adverse changes in IR, beta-cell function, and fasting serum glucose [74]. It should be noted that these studies were performed at the cellular level while we measured serum calcium concentrations that are roughly under homeostatic control. Therefore, its serum change might be minimal as shown in our results. Moreover, blood calcium level is not a good indicator because it is strongly regulated. Only in extreme conditions, such as severe hyperparathyroidism or malnutrition, the serum ionized calcium level is above or below the normal range.

As expected, in our study, serum TC and TG levels were significantly higher in subjects with MetS (Table 2). The prominent role of dyslipidemia in developing CKD has been confirmed in numerous studies. In a study among 2,380 American Indians, Lucove et al. reported a positive association between MetS occurrence and elevated CKD risk after ten years of follow-up [75]. In another study by Ninomiya et al., after five years of followup in 1,440 Japanese adults, 10.6% of participants with MetS developed CKD [76]. In our study, we also observed positive associations between renal function tests and serum lipid profile that confirm previous findings (Table 4). These associations were diverse between groups; for example, albumin was in positive association with TG in individuals with obesity and MetS and combination analysis, while serum urea and uric acid were positively associated with WC in individuals with obesity and without MetS and in combination analysis. Ming et al. [77] also revealed that large WC is related to CKD to a less extent after adjusting the components of MetS.

Similarly, other studies reported positive associations between MetS components and renal function tests. For example, in a study by Wang et al., serum TG level was associated with reduced renal function only in patients with T2DM [78, 79]. In the current study, serum creatinine was non-significantly higher among individuals with MetS, which was partially in accordance with the findings of a previous study that reported a negative relationship between creatinine clearance and the prevalence of MetS [80]. In another study, a high LDL/HDL ratio was associated with elevated serum creatinine values [81]. The exact mechanism explaining the association between MetS and renal disease has not been completely clarified; however, the suggested pathophysiological factors include IR, oxidative stress, endothelial dysfunction, hyperfiltration, and renin–angiotensin–aldosterone-system activation [82]. A prior study by Lee et al. revealed that early intervention in MetS could make the CKD progression slower in patients with early-stage CKD, and help to recognize the risk of MetS change on kidney function in patients [82].

The observed results might be due to the role of IR in the induction of oxidative stress and inflammation and consequently reduced renal function [83]. IR exacerbates kidney disease through such mechanisms as sodium maintenance, increased glomerular filtration rate (GFR), and reduced Na^+^, K^+^- ATPase activity [84, 85]. IR can also cause excessive production of very low-density lipoprotein (VLDL) and the development of hypertriglyceridemia [86].

### Limitations of the current study

This study has some limitations; first of all, the assessment of renal function tests was limited, and no information on GFR, and blood electrolytes was available. Second, since the sample size was relatively small, the findings should be generalized with caution. So, large-scale prospective studies are still required to confirm the findings. Third, the effect of dietary intake on the relationship between MetS and renal function tests was not considered and we lacked data on mineral consumption from the diet. Finally, because this was a case–control study, there was a possibility of confounding and recall bias. However, the strengths of the current study should also be mentioned, it is the first study that emphasized the possible role of some important micro-element and kidney function tests in the pathogenesis of obesity-related disorders and MetS. These findings have some clinical importance; first, the results of the current study suggest that change in mineral status could be a good prognostic biomarker in obesity and its related comorbidities like CVD, T2DM and CKD that could be used in clinical practice. Moreover, from the clinical point of view, chronic disturbed mineral status can be a predictor of the development of cardiovascular disease or diabetes in the future. Also, the inverse association can be considered well; in patients with obesity, metabolic syndrome or related diseases, the mineral balance might prevent some adverse effects related to the disease status.

### Future directions

Further longitudinal studies are required to infer the causality. Also, it is suggested that for the future studies, more sensitive and reliable renal function tests be applied and also, other obesity-related conditions like diabetes and cardiovascular disease be examined.

## Conclusion

Our results suggest that disturbed serum lipids in obesity-metabolic syndrome is associated with homeostatic changes in the level of minerals or proteins that are involved in their metabolism. The current findings highlight the possible clinical importance of these elements or proteins as possible prognostic markers in obesity and its related comorbidities. Although, further studies are needed to better explain and clarify the underlying mechanism of observed relationships.

### Electronic supplementary material

Below is the link to the electronic supplementary material.

Supplementary Material 1: Visual analogue scale for appetite measurement

## Data Availability

The datasets used and/or analyzed during the current study are available from the corresponding author on reasonable request.

## List of abbreviations

ATP III: Adult Treatment Panel III
BMI: Body mass index
CAD: Coronary artery disease
CHD: Coronary heart disease
CKD: Chronic kidney disease
DBP: Diastolic blood pressure
ELISA: Enzyme-linked immunosorbent assay
FBS: Fasting blood glucose
GFR: Glomerular filtration rate
HDL: High density lipoprotein cholesterol
LDL: Low density lipoprotein cholesterol
MetS: Metabolic syndrome
SBP: Systolic blood pressure
T2DM: Type 2 diabetes mellitus
TC: Total cholesterol
TG: Triglyceride
VAS: Visual Analogue Scale
WC: Waist circumference
