# Supplementary figures and images for: The association between lipid profile, oxidized LDL and the components of metabolic syndrome with serum mineral status and kidney function in individuals with obesity
Source: BMC Res Notes. 2023 Sep 5;16:196. doi: 10.1186/s13104-023-06472-2 (PMC10481520; doi:10.1186/s13104-023-06472-2)

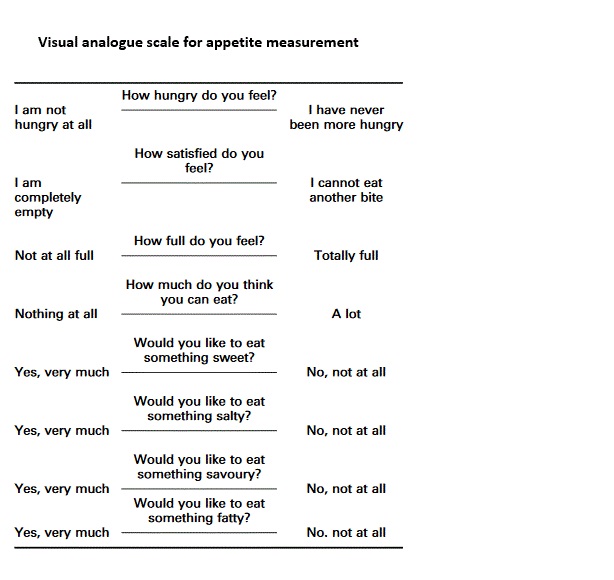

Supplement: Supplementary file 1 — Supplementary Material 1: Visual analogue scale for appetite measurement [file 13104_2023_6472_MOESM1_ESM.jpg]
